# AI in drug discovery and its clinical relevance

**DOI:** 10.1016/j.heliyon.2023.e17575

**Published:** 2023-06-26

**Authors:** Rizwan Qureshi, Muhammad Irfan, Taimoor Muzaffar Gondal, Sheheryar Khan, Jia Wu, Muhammad Usman Hadi, John Heymach, Xiuning Le, Hong Yan, Tanvir Alam

**Affiliations:** a College of Science and Engineering, Hamad Bin Khalifa University, Doha, Qatar; b Faculty of Electrical Engineering, Ghulam Ishaq Khan Institute of Engineering Sciences and Technology, Swabi, Pakistan; c Faculty of Engineering and Technology, Superior University, Lahore, 54000, Pakistan; d School of Professional Education & Executive Development, The Hong Kong Polytechnic University, Hong Kong; e Department of Imaging Physics, MD Anderson Cancer Center, The University of Texas, Houston, USA; f School of Engineering, Ulster University, Belfast, United Kingdom; g Department of Thoracic Head and Neck Medical Oncology, Division of Cancer Medicine, The University of Texas, MD Anderson Cancer Center, Houston, USA; h Department of Electrical Engineering, City University of Hong Kong, Kowloon, Hong Kong

**Keywords:** Artificial intelligence, Biotechnology, Graph neural networks, Molecule representation, Reinforcement learning, Drug discovery, Molecular dynamics simulation

## Abstract

The COVID-19 pandemic has emphasized the need for novel drug discovery process. However, the journey from conceptualizing a drug to its eventual implementation in clinical settings is a long, complex, and expensive process, with many potential points of failure. Over the past decade, a vast growth in medical information has coincided with advances in computational hardware (cloud computing, GPUs, and TPUs) and the rise of deep learning. Medical data generated from large molecular screening profiles, personal health or pathology records, and public health organizations could benefit from analysis by Artificial Intelligence (AI) approaches to speed up and prevent failures in the drug discovery pipeline. We present applications of AI at various stages of drug discovery pipelines, including the inherently computational approaches of *de novo* design and prediction of a drug's likely properties. Open-source databases and AI-based software tools that facilitate drug design are discussed along with their associated problems of molecule representation, data collection, complexity, labeling, and disparities among labels. How contemporary AI methods, such as graph neural networks, reinforcement learning, and generated models, along with structure-based methods, (i.e., molecular dynamics simulations and molecular docking) can contribute to drug discovery applications and analysis of drug responses is also explored. Finally, recent developments and investments in AI-based start-up companies for biotechnology, drug design and their current progress, hopes and promotions are discussed in this article.

## 1 Introduction

Six to seven% of global gross domestic product (8.5 to 9 trillion US$) is spent on healthcare annually [1] and bringing a new medicine to market costs well over $1 billion and can take up to 14 years [2]. Success in drug development (defined as phase I clinical trials to drug approval) is very low across all therapeutic categories worldwide [3] with, for example, 97% of the cancer drugs failing during clinical trials [4]. This makes investments risky and inflates the price of approved drugs to compensate for all the failures [5].

With the digitization of medical records; clinical trials, precision medicine, drug discovery, and health policy will be able to benefit from data-driven methods. Drug discovery has been radically transformed over the last decade by such novel analytical methods and computational advances [6] [7] [8] [9] [10]. Due to recent progress, there is a great interest in the application of artificial intelligence (AI) methods to improve various stages of drug discovery pipeline, including *de novo* molecular design and optimization, structure-based drug design, and pre-clinical and clinical development [11]. Biomedical datasets, such as genomic profiles, imaging data, and chemical and drug databases, can be coupled with analytical methods, especially deep learning models, to coordinate the tools needed to discover useful drugs and their clinical applications [12].

**Motivation for this survey:** Multiple reviews are available on the application of AI in drug discovery. For example, the role of GPU computing and deep learning models for drug discovery is presented in [13], deep learning for precision medicine in [14], generative models for calculating the electronic properties of materials in [15], the advancements due to the completion of human genome project in [8]. The role of machine learning and its implications in drug discovery for understanding biological interactions is presented in [16]. Methods using 3D structure-based drug discovery and dynamics simulation are covered in [17], and applications of machine learning at various stages of drug design are discussed in [12]. The role of graphs in formulating therapeutic problems as machine learning tasks is presented in [18], the applications of AI in drug discovery and challenges are highlighted in [19], [20], [21]. In [22], the progress of traditional machine learning algorithms for protein-ligand docking scoring functions, and in [23] machine learning-based scoring function for structure-based virtual screening are presented.

In this review, we discuss the challenges involved in data representation and prediction, which are key problems in drug design, and where AI can excel. Many drug discovery tasks are difficult to formulate as machine learning problems, due to a lack of AI-ready benchmark datasets and standardized knowledge representations. For example, drugs can be represented in a number of different formats; such as SMILES strings, extended connectivity fingerprint (ECFP), and graphs. Similarly, protein can be represented as 1D amino acid representation, protein sequence representation, and 3D-structure. Another problem is the low resource labels and disparity among labels to formulate meaningful learning tasks. We also discuss the potential use of machine learning libraries, different molecular representations, and the role of graph neural networks at different stages of the drug discovery pipeline, as well as problems in data collection, labeling, disparity among labels, small sample size, noisy labels and approaches to deal with them. The last two years have seen great progress in utilizing deep-learning methods for drug discovery. Many open-source tools [24], AI-ready benchmark datasets [25] and deep learning platforms [26], tailored for drug design have been developed. We present updated and in-depth insights on these topics.

**Figure 1:**
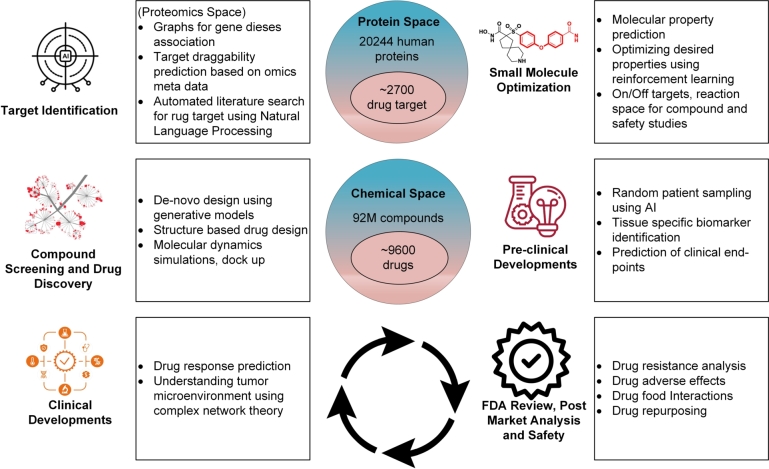
Applications of AI-based methods at different stages of a drug discovery pipeline. There are about 2700 known potential drug target proteins in the human body and about 9600 FDA-approved small molecule drugs [27], [28], [29]. Machine learning can be used to identify the targeted protein, GNNs can be used for predicting drug-target interactions and binding affinity, and reinforcement learning can be used to optimize the properties of a molecule. Computer vision can determine the spatial state of the tumor microenvironment. Generative models can be employed to design new molecules, simulation-based studies can suggest properties of protein-drug complexes, such as stability and dynamics, and NLP can be used to mine the existing scientific literature for drug re-purposing, FDA review, and post-market analysis.

A drug discovery pipeline will usually consist of several stages as shown in Fig. 1. In target-based discovery, the first step is to identify novel targets, with evidence of association to disease, from a large space of proteins (an organism's proteome) [12]. Potentially interacting molecules are identified by high throughput screening of compound libraries against these targets. Compounds will be optimized for favorable drug properties, tested in pre-clinical and clinical trials, and given FDA approval in the ideal case. All stages of the drug discovery pipeline could benefit from AI [11], for example, generative models for the design of new synthetic molecules [30], reinforcement learning (RL) to optimize properties of molecules in a particular direction [31], GNNs to predict drug-disease associations, drug-repurposing, and the response to a drug [32]. Natural language processing (NLP) could be used to find drugs by mining the scientific literature and to automate FDA approval steps [33], [34]. These applications of data science to drug discovery are discussed in (Section 2).

Predicting the three-dimensional structures of potential target proteins, solely from their amino acid sequence, is often necessary for drug discovery, and AI systems had a major recent success in this, with AlphaFold2 [35] winning the Critical Assessment of Structure Prediction CASP14 [36]. Existing deep learning-based libraries, such as DeepChem and DeepAffinity, and databases, including PubChem, PDB, and ChEMBL, that could help drug discovery are discussed, along with AlphaFold2, in Section 3.

As drug discovery applications focus on the three-dimensional structures of molecules (proteins, DNA, RNA, and drugs/medicines) and their interactions, the atom is the fundamental unit of these structures and can be considered as a “machine learning datatype”. Molecular systems contain poorly described higher-level patterns, which could be learned from their data. Interrelations among biomedical data are attributes that could be represented in the form of graphs in the design of data-driven systems. Graph machine learning allows modeling of unstructured multimodal datasets [37] and so could model more complex relationships between drugs and disease, protein-protein interactions, side effects of drugs, prediction of responses to a drug and drug re-purposing [18]. When coupled with an attention mechanism, graph machine learning may identify drug binding sites [38], highly communicating residues/atoms, and provide more interpretable models [39]. A detailed discussion of molecular representation, GNNs, and their application in the context of drug discovery processes is presented in Section 4.

Experimental high-throughput screening, combinatorial chemistry, and other technical methods have been the main choices to create new chemical entities with specific desired features [40] but AI applications now have the potential to be better than a human expert [41]. The application of GNN, generative models and RL for *de novo* molecule generation and optimization is presented in Section 5.

Simulation of bio-molecular structures by detailed, physics-based atomic methods, such as molecular dynamics (MD), [17] is central to drug discovery and biotechnology. The 3D structures of proteins and drugs from the Protein Data Bank (PDB) and DrugBank (or structures predicted by AlphaFold2) can be docked for MD simulations, to investigate the stability, dynamics, geometry, and binding efficacy of a protein-drug complex, giving a time-trajectory of atomic movements. Deep learning or advanced data analysis methods can be applied to analyze these trajectories of biological systems [42], hopefully leading to new hypotheses about the structural changes and interactions in complex biological systems, that may answer questions about diseases, pathways, and drug-response / resistance mechanisms. Structure-based drug design, with the application of MD simulations, for the analysis of drug response and resistance, is discussed in Section 6.

The interests of big pharmaceutical and start-up companies in using AI for drug development, are highlighted in Section 7. Current challenges, and what can be expected in the near future are presented in (Section 8), with conclusions in Section 9. Nano-medicine, medical robotics, medical imaging, and multi-omics data analysis are beyond the scope of this review. The terms small molecule and drug are used interchangeably throughout the manuscript.

## 2 Application of data science in the drug discovery process

The emergence of epidemics and pandemics, such as influenza and COVID-19 [43], and the prevalence of severe diseases, such as cancer and heart disease, demonstrate the ongoing need to discover new drugs. A multi-stage process (Fig. 1), requiring target identification, validation, high throughput screening, animal studies, safety and efficacy protocols, clinical trials, and regulatory approval, is usually followed [12]. Development of a new drug takes approximately 14.6 years and costs about US$ 2.6 billion [2] on average. AI-based methods could be utilized at several stages in this process: identifying novel targets [44], evaluating drug-target interactions [45], [46], examining disease mechanisms [12], and improving small molecule compound design and optimization [47]. These methods can also be used to identify and develop prognostic bio-markers, and study drug efficacy, response, and resistance [132].

### 2.1 Target identification in drug discovery

Target identification during drug discovery aims to identify molecules, usually proteins, that could alter a disease state if their activity was modulated. Machine learning algorithms can analyze various types of data, including gene expression profiles, protein-protein interaction networks, and genomic and proteomic data, to identify potential targets that are likely to be involved in disease pathways [48]. Of the approximately 20,000 proteins in the human proteome, only about 3,000 have been identified as potential therapeutic targets [49]. Future knowledge might expand our understanding of which proteins could become drug targets.

The first step in identifying a target is to establish a causal relationship between the target and the disease [50]. Causal relationships between genes and diseases can be identified using graphs, GNNs, or tree-based methods. A decision tree-based meta-classifier trained on a network topology involving protein-protein, metabolic, and transcriptions interactions, and tissue expression and sub-cellular localization of proteins was proposed in [51] to predict morbidity-associated genes that are also druggable. Regulation by multiple transcription factors (TFs), centrality in metabolic pathways, and extracellular location were identified as key parameters from the decision tree. Machine learning-based methods classified proteins as drug targets or non-targets for specific diseases, such as lung, pancreatic, and ovarian cancer, based on features such as protein-protein interaction, gene expression, DNA copy number, and occurrence of mutations [44].

The primary source of information on target association with disease is the literature. Text mining and Natural Language Processing (NLP) approaches can also be used to identify relevant target-disease pairs from literature and develop databases for target identification [52]. BeFree [53], PKDE4J [54] and other deep learning-based tools [55] can be used to mine articles to identify drug–disease, gene–disease, and target–drug associations.

Drug–target interactions may also be inferred, based on descriptor similarity to reference ligands, in the same cell without explicitly addressing the target identity of those reference ligands. A software tool (SPiDER) [56] discretizes the input feature similarity vector onto a so-called feature map using a neural network-inspired approach.

### 2.2 Virtual screening and optimization of compounds

AI can be used to virtually screen and optimize compounds, estimate their bio-activities, and predict protein-drug interactions [57]. One way AI can help in virtual screening is through the development of predictive models, that can identify compounds with a high probability of binding to a target protein. These models can be trained using various types of data, such as known protein-ligand complexes, structural information, and molecular descriptors. Physico-chemical properties of the drug, such as solubility, partition coefficient (logP), degree of ionization, and intrinsic permeability, may have an indirect effect on a drug's interaction with a target receptor family and must be considered when designing a new drug [58]. AI can also be used to plan efficient routes for chemical synthesis and develop insights into the reaction mechanisms of drugs to identify potentially unwanted interactions with other molecules.

Candidate structures of drugs are refined and modified to improve target specificity and selectivity, and their pharmacodynamics, pharmacokinetics, and toxicological properties. A virtual chemical space with structure and ligand information may provide profile analysis, faster elimination of non-lead structures, and speed up the drug discovery process by avoiding costly time-consuming laboratory work. Multi-objective optimization methods can tune molecules in a desired direction [47]. MD simulation and docking methods can be used to model the orientation, stability, and dynamics of the compounds.

### 2.3 Pre-clinical and clinical development

Predicting possible responses to a drug is a critical step in a drug design pipeline. Similarity or feature-based machine learning methods can be used to predict the response of a drug on individual cells and the efficacy of a drug-target interaction by binding affinity or free energy of binding. Similarity methods assume that similar drugs act on similar targets [59], while feature-based methods find individual features of drugs and targets and feed the drug-target feature vector to the classifier. Deep learning-based methods, such as DeepConv-DTI [45] and DeepAffinity [38] are examples methods, where the embedding of drugs and targets are learned using convolution and attention mechanism.

AI-based techniques can assist in selecting potential patients for pre-clinical trials by identifying relevant human-disease bio-markers and anticipating potential toxic or unnecessary side effects [60] and by filtering a high dimensional set of clinical variables to select a cohort of patients. AI can also help in predicting the outcome of clinical trials well ahead of the actual trial minimizing the chance of any harmful effect on patients [61].

### 2.4 FDA approval and post-market analysis

Natural Language Processing (NLP) can be used to mine scientific literature to report adverse effects, such as toxicity, of a drug or resistance to it and prepare automated evaluations for regulatory (FDA) approval or a patent application [62]. NLP-based sentiment analysis methods can be used to recommend drugs [63]. Prediction of likely sales of a product by machine learning-based systems could help pharmaceutical companies optimize their business resources [64].

## 3 Existing databases and tools for drug development

### 3.1 Chemical and biological databases

Experimental bio-assay and computationally produced drug-target interactions (DTI) data need to be collated in publicly available databases. Compound and bio-activity databases are listed in Table 1 and target and chemical databases are given in Table 2.

**Table 1:** List of Compound and Bio-activity databases.

Reference	Description	Link
PubChem [27]	Largest collection of freely accessible chemical and bio-activity information	https://pubchem.ncbi.nlm.nih.gov/
ChEMBL [65]	A large-scale bioactivity database for drug discovery	https://www.ebi.ac.uk/chembl/
DrugBank [66]	A knowledge-base of drugs, drug actions, and drug targets	https://go.drugbank.com/
ZINC [67]	An open resource for virtual screening of compounds	https://zinc.docking.org/
BindingDB [68]	A database of measuring binding affinity between target and the drug	https://www.bindingdb.org/bind/index.jsp
ADME [69]	An online database for pharmacokinetic information	https://www.fujitsu.com/global/solutions/business-technology/tc/sol/admedatabase/
STITCH [70]	An integrated database of chemical-protein interactions	http://stitch.embl.de/
SIDER [71]	Marketed medicines and their recorded adverse drug reactions	http://sideeffects.embl.de/
GDSC [72]	Drug response data and genomic biomarkers	https://www.cancerrxgene.org/
PDBBind [73]	A comprehensive collection of binding affinities for the protein–ligand complexes in the Protein Data Bank (PDB)	http://www.pdbbind.org.cn/
canSar [74]	Cancer translational research and drug discovery knowledgebase	https://cansarblack.icr.ac.uk/

**Table 2:** List of Target and Chemical databases.

Reference	Description	Link
PDB [75]	Protein data bank archive provides information about 3D structure of protein, nucleic acids and complex assemblies	https://www.rcsb.org/
UniProt [28]	An open resource of protein sequences and functional information	https://www.uniprot.org/
Atom3D [76]	A benchmark of existing datasets of 3D molecules, spanning on several types	https://github.com/drorlab/atom3d
TTD [77]	A therapeutic target database	http://db.idrblab.net/ttd/
MoleculeNet [78]	A benchmark of datasets for molecular machine learning	https://moleculenet.org/

#### 3.1.1 PubChem

PubChem [27] is the largest free database of chemical information, with about 111 Million compounds, 279 Million substances, 295 Million bio-activities, and 34 Million articles, organized into three inter-linked web data pages; substance, compound, and bio-assay [79]. The descriptions of, and test results from, bio-assays are stored in the bio-assay database. Data mining methods can be used to identify compounds for a particular target or protein.

#### 3.1.2 ChEMBL

ChEMBL [65] is an open-access drug discovery database, developed by the European Molecular Biology Laboratory (EMBL). Data on authorized and candidate medications, such as the mechanism of action and therapeutic indications, are gathered from full-text papers in high-impact publications and combined with data on small, compounds and their biological activity. The bio-activity data is exchanged with another database; such as BindingDB [68] and PubChem Bioassay. The ChEMBL database has been used to identify chemical tools for a target of interest, to predict drug-target interactions, to re-purpose a drug, to determine target tractability, and to integrate with existing drug discovery tools [29].

#### 3.1.3 DrugBank

DrugBank provides molecular-level data, clinical information, drug interactions, side effects, and drug re-purposing. It is widely used for *in silico* drug design, re-purposing, and drug discovery using machine learning.

#### 3.1.4 UniProt database

UniProt [28] is a public database of protein sequences annotated with taxonomic data and information on biological functions. There are four components; UniProt Knowledgebase (UniProtKB), UniProt Reference Clusters (UniRef), UniProt Archive (UniParc), and UniProt Metagenomic and Environmental Sequences (UniMES). Uniprot contains more than 189 million records; more than half were curated by human experts.

#### 3.1.5 Protein data bank

The Protein Data Bank (PDB) is the largest database of the 3D-structures of proteins, ribosomes, and nucleic acids that were determined primarily by X-ray crystallography or nuclear magnetic resonance spectroscopy [75].

### 3.2 AI-based software tools for drug development process

AI tools have the potential to transform drug discovery by enabling researchers to rapidly analyze large-scale data sets, design new molecules, and predict the efficacy of potential drug candidates. Here, we review some of the popular AI tools for drug discovery applications.

#### 3.2.1 AlphaFold2

Predicting the 3D structures of proteins from their amino acid sequence is a very complex and challenging problem. AlphaFold2, developed by DeepMind, has achieved a breakthrough level of accuracy [35]) and is openly available via Google Colab.

#### 3.2.2 DeepChem

The DeepChem [80] library is a Tensorflow wrapper that understands and streamlines the analysis of chemical datasets. It has been used for algorithmic research into one-shot deep-learning algorithms for drug discovery and application projects such as modeling inhibitors for BACE-1) [80], [90]. DeepChem can be used to analyze protein structures, predict the solubility of small molecule drugs and their binding affinity to targets, and count the number of cells in a microscopic image. MoleculeNet [78], which contains the properties of 700,000 compounds has been integrated into the DeepChem package.

#### 3.2.3 DeeperBind

DeeperBind [81] is a long short-term recurrent convolutional network that predicts protein binding specificity in relation to DNA probes, which can model the interaction between transcription factors (TF) and their corresponding (DNA/RNA) binding sites. DeeperBind can effectively predict the dynamics of probe sequences. It can also be trained and tested on datasets with sequences of variable lengths.

#### 3.2.4 DeepAffnity

DeepAffinity [38] is a semi-supervised model that unifies recurrent and convolutional neural networks to predict the binding affinity between a drug and target sequences. The model uses both labeled and unlabeled data to jointly encode molecular representations under unique structurally annotated protein sequence representations. DeepAffinity outperformed random forest, ensemble methods, and RNN-CNN models. A list of AI-based software for drug discovery is given in Table 3.

**Table 3:** List of AI-based software for drug discovery, development, and analysis.

Reference	Description	Source code
AlphaFold2 [35]	Deep learning based model for 3D structure prediction of proteins from amino acid sequences	https://github.com/deepmind/alphafold/
DeepChem [80]	A deep learning library for drug discovery and computational chemistry	https://github.com/deepchem/deepchem
DeepBind [81]	A computational tool to analyze binding between the protein and DNA/RNA	https://github.com/MedChaabane/DeepBind-with-PyTorch
DeepBar [82]	A method for accurate and fast prediction of binding free energy	https://fastmbar.readthedocs.io/en/latest/
Deep-Screening [83]	Web-server based in deep learning for virtual screening of compounds	http://deepscreening.xielab.net/
DeepScreen [84]	High performance drug target interaction	https://github.com/cansyl/DEEPScreen
DeepConv-DTI [45]	A convolutional neural network based model for predicting drug-target interactions	https://github.com/GIST-CSBL/DeepConv-DTI
DeepPurpose [24]	A Deep learning library for drug-target interaction, drug-drug interaction, protein-protein interaction and protein function prediction	https://github.com/kexinhuang12345/DeepPurpose
DeepTox [85]	A deep learning model for toxicity prediction of chemical compounds	http://www.bioinf.jku.at/research/DeepTox/
AtomNet [86]	A deep convolutional neural network for bioactivity prediction	github
PathDSP [87]	A deep learning method for predicting drug sensitivity using cancer cell lines	https://github.com/TangYiChing/PathDSP
Graph level representation [88]	Learning graph representation for drug discovery	https://github.com/ZJULearning/graph_level_drug_discovery
Chemical VAE [89]	An auto-encoder based framework to generate new molecules	https://github.com/aspuru-guzik-group/chemical_vae/
DeepGraphMol [87]	A computational method for molecule generation with desired properties using graph neural networks and reinforcement learning	https://github.com/dbkgroup/prop_gen
TorchDrug [26]	A pytorch based flexible framework for drug discovery models	https://torchdrug.ai/

## 4 Data representation and graph neural networks for drug discovery applications

Machine-readable representations of molecules allow rapid computing, querying, and storage of molecules in machine learning algorithms for drug discovery [91]. Their quality can affect the utilization of the variation in the data [92].

Most machine learning algorithms assume both training and testing data are independent and identically distributed [93], However, this assumption does not hold valid for drug discovery applications. Small molecule optimization and design necessitate the exploration of structural variations drawn from purposely unique chemical space. A model must generalize to out-of-distribution situations in order to be useful. Despite the distribution shift, chemo-informatics and medicinal chemistry will benefit from learned features [91]. Here, we discuss some key advancements in molecular representation learning.

### 4.1 Molecule representations

**Figure 2:**
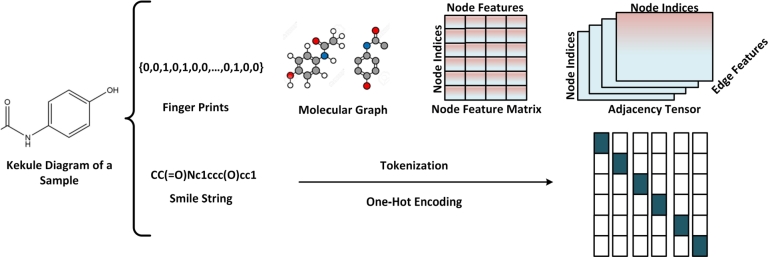
Illustration of different formats of Small Molecule Representations. Molecules can be represented as Kekule diagrams with bonds and atoms, SMILES strings (which can be converted into a one-hot encoding), and as molecular graphs, where adjacency, node, and feature matrices can be constructed.

Fixed molecular descriptors can be classified based on their dimension [94]. Molecules have 0D attributes, such as molecular weight (MW), atom number, and atom-type count. For functional groups, descriptors involving more structural information are needed, such as fingerprints (two-dimensional binary vectors) [95]. More complex representations, such as SMILES [96], molecular graphs, and fingerprints [97], were developed for machine learning algorithms (Fig. 2).

Molecular descriptors used in machine learning models [58] are fixed and not learnable. String-based representations, such as SMILES [96] that are widely used for storage in chemical databases [27] compactly encode molecular structure. SMILES is a line notation that uses short ASCII strings to describe the structure of chemical species and can be converted into a one-hot encoding or word embedding for machine learning and NLP methods. A review of different representations for bio-molecules is presented in [98]. The paper presents [98] atom-based, residue-based, and graph-based representations, and also highlight the importance of choosing an appropriate representation for the specific problem at hand, and notes that combining different types of representations can lead to more accurate and effective models.

For deep learning, compounds, and target can be represented in different encodings; for example, we can use Transformer encoders to learn SMILES representations and Recurrent Neural Networks (RNN) for protein representations. Molecules can also be embedded directly into the continuous latent space, without feature engineering, by a molecular graph (G = (V, E)) [97] where atoms or residues are mapped to nodes (V) and bonds or connections between nodes are assigned to edges (E). The attributes of each atom can be represented by a node matrix *X* and those of bonds are represented by an edge matrix *E*, while an adjacency matrix *A* can keep a record of the pairwise connections. An adjacency tensor is usually formed by combining the edge feature matrix with the adjacency matrix. The graph representations allow more structural information to represent a molecule.

### 4.2 Topological data analysis

Topological data analysis (TDA) [99] can be used to examine complex data sets, such as the representation of biomolecules. TDA is based on Algebraic topology. a branch of mathematics that examines the characteristics of spaces that are preserved through continuous transformations, serves as the foundation for TDA [100]. In [101] proposed algebraic topology, specifically persistent homology, to extract topological features from molecular structures in order to overcome this limitation. Their analysis reveals that the machine learning model performs better when persistent homology features are added to predict binding affinity and identify active compounds in virtual screening.

A novel representation of bio-molecules based on their underlying topology, which captures the shape and connectivity of atoms in a molecule is proposed in [102]. Deep convolutional neural networks (CNNs) are trained to learn a hierarchical representation of the molecule's topology, to predict various properties of the molecule. The multi-task learning framework is used, to predict several molecular properties at once. This method can increase the accuracy of predictions for specific properties because it makes use of shared representations across various tasks.

#### 4.2.1 Topological data analysis for protein-ligand binding affinity prediction

One typical method to predict the protein-ligand binding affinities of a compound is to visualize the protein-ligand complex as a persistent diagram, which is a geometric object. The complex's topology, including the number of connected components and the existence of holes and voids, is depicted in the persistence diagram. In [103], authors proposed persistent homology to extract features from the complex geometries of protein-ligand complexes. These features are then used as inputs to a machine learning algorithm, trained to predict the binding affinity of new protein-ligand complexes. The approach is called PerSpect ML, which stands for “Persistent spectral-based machine learning.” The authors demonstrate that PerSpect ML outperforms existing state-of-the-art methods for protein-ligand binding affinity prediction on several benchmark datasets.

Wee et al. [104] proposed a combination of persistent homology and machine learning for binding affinity prediction. The approach is based on Forman's Ricci curvature, a geometric quantity that characterizes the local geometry of space and is a useful tool in mathematics. The Forman persistent Ricci curvature (FPRC), is a variation of Forman's Ricci curvature, used to extract topological features from the intricate geometries of protein-ligand complexes. The findings demonstrate the efficacy of the proposed approach for drug development.

The use of hypergraph, persistent homology, and machine learning to predict the binding affinity is presented in [105]. In order to capture the topological and geometric characteristics of the complex, the authors used persistent homology to extract features from the hypergraph and fed it to a machine learning model. The proposed “PSH-ML” outperforms current state-of-the-art approaches for predicting protein-ligand binding affinity on several benchmark datasets.

### 4.3 Graph neural network

Most biomedical data, such as protein-protein interactions, protein-drug interactions, drug-disease interactions, and drug-repurposing used in drug discovery is interconnected and so suitable to be represented by a graph. Small molecule drugs can also be represented as graphs, with atoms as nodes and chemical bonds as edges. Knowledge graphs can be used to present complex relationships between drugs, adverse effects, drug re-purposing, and associated outcomes to assist in generating novel hypotheses.

An important structural attribute of a graph is that nodes are usually not required to be presented in any particular order, and functions acting on graphs should be permutation invariant (order-independent) so that the output of those functions should be the same for any two isomorphic graphs. This property makes a graph a suitable candidate to represent molecules and drugs. Molecular graphs and subgraphs can be readily mapped to a chemical (sub-)structure, making them interpretable.

**Figure 3:**
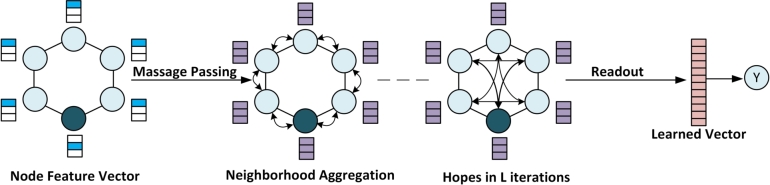
Graph Neural Networks in Prediction Mode. Molecules can be represented as linear data structures, such as adjacency, node, or feature matrices. These matrices can be fed to graph neural networks to learn an embedding, which can be used to predict molecular properties.

Graph neural networks (GNNs) are a type of machine learning algorithm that can be applied to drug discovery. GNNs are designed to work with graph data, which represents relationships between entities, such as chemical compounds and proteins [106]. They encode pairwise connectivity instead of points in a non-Euclidean space, capturing a structured representation of atomistic data. A typical GNN consists of one or more layers that learn a permutation invariant aggregation of nodes from node feature vectors and across the neighboring nodes [107] through recursive message passing, leading to a readout operation (Fig. 3). The concept is that a node in a graph constantly exchanges information/messages with its neighbors until it reaches a stable equilibrium.

A feature vector can be constructed by combining different properties of the atoms, such as mass, electron number, and charge. To predict a certain property of a molecule/drug, the spatial structure and feature vectors can be used to learn a meaningful representation. The node feature vectors can be stacked into a matrix X which is multiplied by the adjacency matrix A to capture the underlying structure of the molecules. Increasing the power of the adjacency matrix A to A^*n*^ results in the propagation of features to nodes at an n-hop distance, an effect similar to increasing the receptive fields in images. The learned embedding can be used to predict molecular properties.

GNNs are widely used in drug discovery applications [18]. Directed message-passing GNNs operating on molecular structures were used to offer possibilities to re-purpose drugs as antibiotics [108] and *in vivo* validation gave viable candidates that were structurally unique to existing antibiotics. Other examples are: AlphaFold2 [35] which uses information about proteins to construct a graph of residues; *MolCLR*, a self-supervised method to learn molecular representations from a large unlabelled dataset (10 million examples) [109] via GNN encoders that extract useful representations from molecular graphs using graph convolutional (GCN) and graph isomorphism networks (GIN) [110]. The model was fine-tuned using MoleculeNet [78] benchmarks and had an efficient performance on both classification and regression tasks.

#### 4.3.1 Graph convolutional neural networks

Modern graph convolutional networks (GCNs) use customized convolutions and readout functions to learn the common local and global structural patterns of graphs. Each graph's node representations are collapsed into a graph representation via a readout layer. Convolutional graph neural networks (ConvGNNs) [111] generalize the grid-to-graph data convolution technique. Since graph data lies in a non-Euclidean space and there is no fixed input size, the node representation is transformed into a spectral domain using the graph Fourier transform, and the convolution operation is replaced with a simple multiplication.

The idea is to produce a node *v's* representation by combining its own x_*v*_ and neighbors' x_*u*_ characteristics, where *u ϵ N* is the neighbors of *N(v)*. ConvGNNs stack many graph convolutional layers to extract high-level node representations. More complicated GNN models rely on ConvGNNs for their construction; such as spectral-based, spatially based message passing neural networks, graph attention network, and graph isomorphism network (GIN) [112], [113].

#### 4.3.2 Attention-based graph convolution neural networks

Attention networks have become a gold standard when dealing with time series or sequential data. Attention mechanisms allow the network to cope with variable-sized inputs by focusing on the most relevant elements of the information to make decisions. Self-attention [114] or intra-attention is the term used when an attention mechanism is utilized to calculate a representation of a single sequence or a node. In graph attention networks (GAT) [113], the GAT layer extends the GCN layer's basic aggregation function by using attention coefficients to assign varying importance to each edge. In this way, the costly matrix inversion operations are avoided allowing deeper training of the neural networks. GAT mechanisms can be used to identify drug binding sites in a protein-drug complex, highly communicating atoms in a large protein system, and atoms (nodes) involved in predictions of binding affinity [38].

## 5 Deep learning models for molecule generation

**Figure 4:**
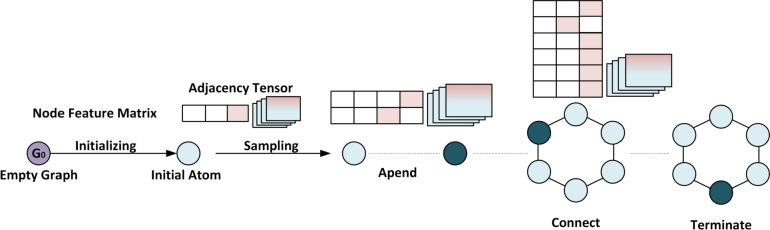
Graph Neural Networks in Generation Mode. Initialization is performed to add the first atom to the empty graph G_0_. A graph transition (append, connect, or terminate) is sampled and performed on the intermediate molecule structure at each step [20].

Graph neural networks (GNNs) can also be used for molecule generation in drug discovery. GNN-based models can generate new molecules with desirable properties by learning the relationships between the atoms and molecular fragments in a given dataset. In MolMP [32], graph creation is modeled as a Markov Decision Process [115] problem, where the action to develop the graph, append, connect or terminate, is only dependent on its current state, with a neural network controlling the sampling process, as shown in Fig. 4. MolMP outperformed SMILES-based molecule creation on a number of different evaluation indicators.

### 5.1 Generative models

Deep molecular generative models allow rapid exploration of a large chemical space [116] including novel structures generated by merging parts of existing compounds. By utilizing genetic algorithms or particle swarm optimization. Generative Adversarial Networks (GAN) [117] can generate synthetic compounds, or molecules, with a desired property and learn the probability distribution of the training data and generate new chemical structures by sampling from the learned probability distribution. Chemical fingerprints, SMILES, molecular graphs, three-dimensional structures, and other molecular representations can be used in generative models. However, assessing the uniqueness, and eventually, the relevance, of the molecules produced by generative models remains an open issue. An Editorial provides guidelines [118] about the assessment of the molecules produced by generative models.

### 5.2 Variational auto-encoder

**Figure 5:**
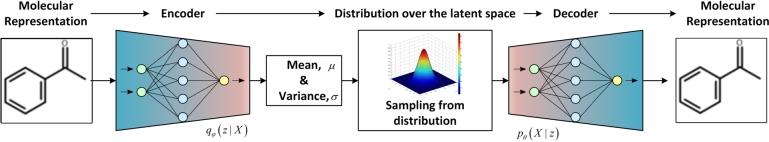
The variational auto-encoder (VAE) for *de novo* design of molecules with desired properties [89]. The neural network converts the discrete input molecule into a Gaussian distribution. The latent variables are reparametrized against the mean and variance. The decoder generates a new molecule from the sampled latent space.

Variational Auto-Encoders (VAEs) were used in [119] to generate novel chemical structures, that were mapped by unsupervised learning into the ZINC database [67] (Fig. 5). The model consists of an encoder, a decoder, and a predictor. VAE converts a discrete molecular structure into a real-valued continuous vector and the decoder converts it back to a discrete structure. The generation of new chemical structures with desired properties can be realized by searching the continuous latent space by any optimization method. The property value is applied to VAE's latent space, which can be used to sample molecules in the direction of the desired property value.

### 5.3 Reinforcement learning

It is difficult to control the properties of generated molecules using continuous data-driven representation [89]. For example, in generative adversarial networks, generating a molecule with the desired set of physio-chemical properties from a large physio-chemical search space is challenging and time-consuming [120]. Reinforcement learning (RL) is a type of machine learning algorithm that has been applied to molecule generation in drug discovery. RL, a machine learning paradigm, which is used to make dynamic decisions, can be used to design chemical compounds with optimal values such as solubility, pharmacokinetic properties, or bioactivity [121]. It entails analyzing potential actions and estimating the statistical relationship between those actions and their potential consequences, then determining a policy that aims to get the best feasible result. Deep reinforcement learning attempts to find the optimal set of actions from the theoretically infinite action space. This property of the algorithm can be exploited for exploring the infinite chemical search space, by avoiding brute-force computing to examine every possible solution. ReLeaSE [122], an RL model for structural evolution, integrates two deep neural networks—generative and predictive. Both networks are trained individually but used together to produce innovative targeted chemical libraries, based on deep RL techniques.

## 6 Structure-based drug design

The completion of the human genome project [14] resulted in the explosion of genomic, proteomic, and structural data. Excellent drug targets are being identified at a faster rate and low cost due to advances in bioinformatics and data analytics methods [123]. Computational structure-based drug design takes advantage of the accumulation of biological data, such as structures of proteins **(Protein Data Bank)** and drug databanks **(DrugBank)**. The knowledge about the potential drug target's structure is extremely valuable, not only for lead discovery and optimization but also in the later stages of drug development, when issues like toxicity, drug resistance, or bio-availability may arise. If experimental structures are not available for a bio-molecule or complex, molecular modeling softwares [124] can be used to predict the structures and their quality can be assessed using computational tools [125]. In this Section, we discuss various methods for structure-based drug design; such as Molecular Dynamics (MD) simulation of the drug-target pair, molecular docking for predicting the orientation, and computational geometry of the drug binding site.

### 6.1 Computational modeling

Although Protein Data Bank (PDB) [75] and DrugBank [66] provide high-quality resources for a large number of protein structures and drug complexes, structural information for a particular drug-target complex may not be available, especially for mutant structures and drug-mutant complexes. In such cases, computational modeling can be used to predict the mutant structures. Rosetta-Commons [124] models protein structures and macromolecular complexes. Other computational and statistical methods ([126], [127]) are available to further assess the quality of the predicted models.

### 6.2 Molecular docking

Molecular docking [128] is used to predict the relative orientations of molecules when they form a complex and allows the estimation of their binding affinity. Several open-source molecular docking software packages, such as Auto-Dock, Flex-Aid, and rDock, are available [129].

Proteins are mobile objects and their ability to make conformational changes influences the protein-drug interactions that molecular docking aims to capture. Molecular dynamics simulations can be used to predict the time-dependent behavior (motion) of protein-drug complexes.

### 6.3 Molecular dynamics simulation

Molecular Dynamics (MD) [130] simulates the movement of molecules such as DNA, proteins, and drug-target complexes. It can be used to identify the free energy landscape and physiological conformations of proteins and complexes, which may even not be accessible through experimental techniques, and so provide insights into the bio-activity of structures and protein-drug complexes. In an MD simulation, the trajectories of all atoms, based on their positions, velocities, and accelerations are obtained using Newton's second law of motion. MD simulations are computationally expensive and require effective computational resources, such as parallel computing.

MD simulation packages, such as Amber, Gromacs, and Charmm [131], provide functions to analyze, visualize and predict the properties of proteins, drugs, and complexes. Table 4 provides a list of computational tools for MD simulation packages.

**Table 4:** List of software for MD simulation, Modeling, Docking, Visualization and analysis of Molecules.

Reference	Description	Pros	Cons	Source code
AMBER [144]	A package for MD simulation	High Performance MD, Comprehensive trajectory analysis tools	License required for parallel CPU or GPU computation	https://ambermd.org/
ACEMD [145]	An accelerated platform for faster and longer biomolecular simulations	Super computer level performance	License required for ful functionality	https://www.acellera.com/
AutoDock Vina [146]	A program for molecular docking and screening	Receptor flexibility, blind docking	Difficult to dock small peptides	https://vina.scripps.edu/
DeePMD [147]	A deep learning package for MD simulation and energy representation	Optimized code, interfaced with Tensorflow	Model compression issues	https://github.com/deepmodeling/deepmd-kit/
RBio3D [148]	R package for the analysis of MD trajectories	Tools for protein-networks, conformations	-	http://thegrantlab.org/bio3d/
Pymol [149]	An interactive platform for visualization of molecules	Homology Modeling, Docking, Virtual Screening	License required for full features	https://pymol.org/2/
RosettaCommons [124]	A tool for predicting the mutant structure	Protein modeling and folding	Preference for aromatics, Preference for hydrogen bonding	https://www.rosettacommons.org/

**Figure 6:**
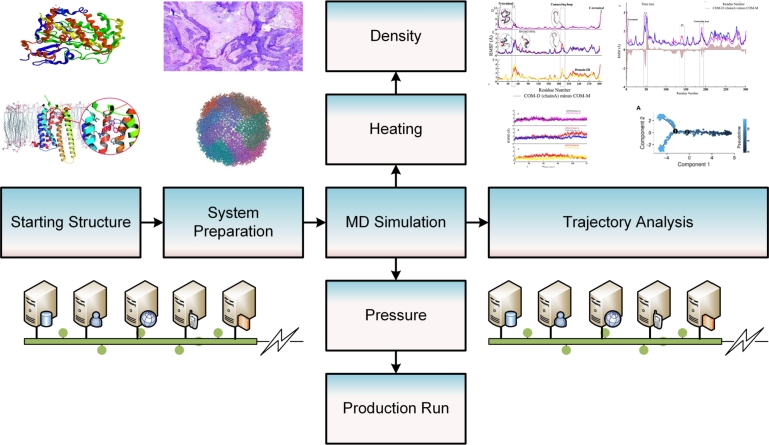
A pipeline for a molecular dynamics simulation. The MD simulation pipeline can be divided into three steps, (i) System preparation, including solvation and topology and coordinate file generation (ii) System simulation for the desired time scale (iii) System or trajectory analysis using analytical methods.

Fig. 6 shows a pipeline for performing MD simulations. Starting from the template structure, the structure is solvated by a water box, and a molecular force field is selected. The system is neutralized, energy minimized, heated, and equilibrated before a production run is performed, usually over several nanoseconds.

Root mean square deviation is commonly used to analyze the fluctuation between different ‘snapshots’ or time points in the MD trajectory [132]. Binding free energy can be used to estimate the strength of binding between a drug and target over time and principal component analysis can be used to analyze the dominant motions [133]. Network theory can be applied to extract different conformational communities [134]. The stability of a structure can be analyzed using correlation [135] and hydrogen bond analysis [136]. The impact of mutations on drug binding affinity can be estimated using time series or geometrical properties of the complex [137]. Machine learning-based models can be used to predict the drug response based on features extracted from the MD simulation [138].

#### 6.3.1 Machine learning-based MD simulation models for drug discovery applications

Molecular dynamics simulation calculations can be sped up using machine learning techniques. To accelerate the simulation towards more energetically advantageous states, for instance, machine learning models can be trained to predict the potential energy of a given configuration of atoms. This strategy is referred to as “machine learning force field” or “machine learning potential [139]”.

As mentioned above, machine learning is currently taking on a more and more crucial role in Structure-based drug discovery. Researchers can now understand the binding mode, affinity, and evolution of atomic systems by using appropriate models and algorithms that allow the chosen model to “learn” the patterns present in the input data. This is especially true of advances made in the use of DL-based MD computational methods [140].

### 6.4 Modeling the binding pocket of a protein-drug complex

The energy released due to bond formation and protein-ligand interactions is known as the free energy of binding and it can be used to estimate the binding affinity and predict responses to a drug. The MD trajectory and the (MMGBSA) [141] tool in Amber can be used to calculate the free energy of binding. The energetic contribution of individual residues is used to infer the binding mode of a ligand and protein.

Geometrical features such as the drug binding site position, the number of interacting atoms at the interface of a protein and a drug, and the shape of interacting atoms can be used to evaluate the efficacy of a drug. A drug binding site on a protein will often be a cavity or pocket and so have a concave shape and a greater potential contact surface area and so have a higher molecule affinity than surface protrusions that have a convex shape. The geometry of the complex can be modeled by the Alpha shape [142], which is a linear approximation method that uses geometrical data to reconstruct a target object's surface properties. Alpha shape modeling and Delaunay triangulation methods are used to predict protein-ligand and protein-protein interactions and protein structure [143]. A list of software for molecular dynamics simulation, molecular docking and visualization is given in Table 4.

**Figure 7:**
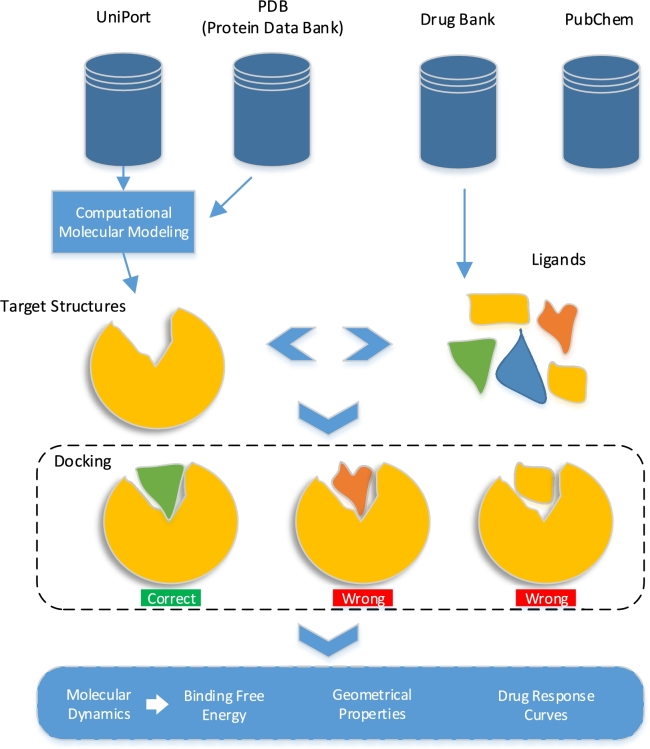
Molecular docking and molecular dynamics simulations can investigate the efficacy of a protein-ligand system, using binding free energy and geometrical properties.

A framework for structure-based docking and drug response analysis is shown in Fig. 7. A target structure from the PDB or other drug databanks or modeling can be used to perform docking followed by MD simulation to investigate the conformations, stability, and binding free energy. Modern geometrical deep learning methods [150] can be used to learn geometry for protein-drug complexes. Drug or drug-dose response curves show the response of an organism or system as a function of exposure to a drug over time, the commonly used parameter is IC_50_ which measures the potency of a substance to inhibit a specific biological or biochemical function. The IC_50_ values are determined by expensive biological experiments and are prone to errors [151]. Deep learning-based methods can be used for the prediction of IC_50_ values [151].

### 6.5 Limitations in current structure-based drug design and a way forward

Time-limitations, inaccuracies in force-fields, quantum effects [17], model interpretation, data collection, and privacy issues [11] affect the ability of MD simulations and molecular docking to provide meaningful information. Biological properties such as protein folding, ligand binding, and release may occur over larger time scales than can be simulated. Selecting (and designing) a correct force field remains a significant challenge and the ability of the force field to mimic reality affects the accuracy of an MD simulation.

Classical MD simulations are incapable of simulating the chemical reaction of a drug substrate as well as the binding of covalently bonded ligands. Electronic polarization and the quantum effect are difficult to define in MD simulations.

To model the chemical reactions of a drug substrate, reactive force fields are being developed. Electronic polarization can be modeled by quantum mechanic MD (QM-MD), which is computationally expensive and limited to a small number of atoms. The high arithmetic and inherent parallelism of graphical processing units (GPUs) can be used to run longer MD simulations.

Overall, the use of machine learning in molecular dynamics simulations has the potential to significantly speed up the discovery of new substances and molecules with desirable properties, such as enhanced catalytic activity or improved drug binding.

## 7 AI-based pharmaceutical start-up companies

According to Emersion Insights research, AI start-ups in drug development raised about 2.1 billion USD in the first half of 2021 [152]. AI is already been used by big biopharmaceutical companies at various stages of drug discovery. For example, Pfizer is using IBM Watson, a machine learning-based system to search for immuno-oncology drugs. Roche Gentech is using GNS healthcare from Cambridge, Novartis is using Microsoft for research on cell and image segmentation, and Astrazenecca is associated with BenovalentAI to develop and commercialize Jenssen's novel clinical stage candidates [153].

Companies like Google, DeepMind, Insilico Medicine, Deep Genomics, Healx etc., are also making huge investments in AI-based drug discovery applications. In this Section, we discuss recent developments and prominent AI-based companies for drug development.

USA is the pioneer and the dominant participant in AI implementation and hosting more than half of the world's AI companies for drug discovery businesses. A huge increase in the number of investors in the USA and the European Union has been observed in recent years. As a result, these areas, along with the United Kingdom, are the leaders in terms of the number of investors in AI-based drug discovery applications. Novartis is a major player in the pharmaceutical AI race in the United Kingdom and the European Union. BenevolentAI and AstraZeneca, two UK-based companies, are working together on a novel AI-generated chronic kidney disease target. Recently, China is also focusing on investment in AI for drug discovery and it has vowed to invest US $5 billion in AI. Tianjin, one of China's largest cities, will invest US $16 billion in its AI business, while Beijing will create a $2.12 billion AI development project. By 2030, China envisions becoming the leader in AI-based drug discovery start-ups.

**Figure 8:**
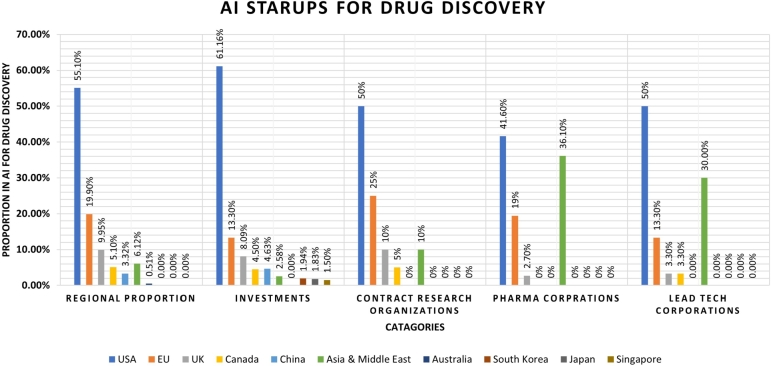
Statistics of AI start-ups for drug discovery.

As shown in Fig. 8, the USA is the leading country with 55.10% companies, followed by Europe and the UK with 19.90% and 9.95% proportion in the adoption of AI-based solutions for drug discovery. Meanwhile, Asia currently has the fourth-lowest proportion in the adoption of AI-empowered drug discovery start-ups [154].

USA also leads the AI race in terms of Contract Research Organizations (CRO), with 50% of CROs situated in the United States followed by Europe which has 25% CROs. Meanwhile, Asia also has 10% CRO interested in AI-oriented drug discovery. According to the number of IT companies using AI in healthcare and drug research, the United States leads all the countries. However, in terms of the number of chemical corporations, Asia has the second highest number, with the EU in third place. This makes sense in light of the EU's recent growth in the chemical sector, which now outnumbers the US and Asian markets for chemical compounds and related goods. Table 5 provides an overview of some major start-up companies using AI to solve industrial problems in pharmaceutical research.

**Table 5:** Overview of top AI-oriented pharmaceutical and biotechnology Start-ups across the globe with major applications in the drug discovery pipeline.

Sr. No.	Company	Country	Year	Major Applications	Revenue/Year	Link
1	Atomwise	USA	2012	Machine learning based discovery of small molecule oriented medicines	17.1M USD	https://www.atomwise.com/
2	Verge Genomics	USA	2015	Drug design for neurodegenrative disease	2.78M USD	https://www.vergegenomics.com/
3	Biovista	USA	1996	Drug re-positioning and de-risking, personalized medicine	4M USD	https://www.biovista.com/
4	Aria Pharmaceuticals	USA	2015	Small molecule design	5M USD	https://ariapharmaceuticals.com/
5	PathAI	USA	2016	Digital pathology analysis for drug development	255M USD	https://www.pathai.com/
6	Recursion Pharmaceuticals	USA	2013	Clinical stage drug development	2.5M USD	https://www.recursion.com/
7	Valohealth	USA	2007	An integrated system for end-to-end drug development	19.4M USD	https://www.valohealth.com//
8	Catalia Health	USA	2014	AI-based platform for remote heath care management	5.9M USD	http://www.cataliahealth.com/
9	Verantos	USA		A real world evidence (RWE) company for clinical, regulatory and reimbursement claims.		https://verantos.com/
10	Insitro	USA	2018	Predictive models for drug development	20.6M USD	https://insitro.com/
11	Trials.ai	USA	2016	Intelligent AI clinical design	1.2M USD	https://www.trials.ai/about-us/
12	ReviveMed	USA	2016	AI-driven drug design for metabolomic diseases	0.26M USD	https://www.revivemed.io/
13	OneThree Biotech	USA	2018	AI-driven drug discovery platform with multiple clinical validations	3.5M USD	https://onethree.bio/
14	BERG Health	USA	2009	Clinical-stage AI-driven biotechnology company	17.9M USD	https://www.berghealth.com/
15	BenevolentAI	UK	2013	Explore inter-connected disease network using data to design effective treatment strategies and drug development.	45.4M USD	https://www.benevolent.com/
16	Nuleome Therapeutics	UK	2019	Decoding dark matter of human genome for new ways of disease treatment	6.3M USD	https://nucleome.com/
17	BioSymetrics	Canada	2015	Phenomics-driven approach for drug discovery	2.6M USD	https://www.biosymetrics.com/
18	Deep Genomics	Canada	2014	AI-based platform for complexities in RNA biology for drug development	9.5M USD	https://www.deepgenomics.com/
19	Insilico Medicine	Hong Kong	2014	AI-assisted identification of drugs	10.9M USD	https://insilico.com/
20	iCarbonX	China	2015	Multi-omics technologies for innovative biomarkers discovery	5M USD	https://www.icarbonx.com/en/

## 8 Challenges, hype, hope, and reality for AI in drug discovery

In the drug-development arena, we have witnessed the rapid change from single molecule design to high-throughput chemical library screening within years; now AI-assisted drug development is on the horizon. In this Section, we discuss the progress of AI on drug discovery applications, challenges in data representation and learning, and discuss the current hype, hope, and reality.

### 8.1 Challenges

Many challenges exist for AI in the drug discovery domain, such as data representation, data labeling, disparity among labels, small sample size, data privacy, ethical concerns, learning paradigms, and model interpretations. For example, a molecule can be represented in a number of ways, such as SMILES, molecular fingerprints, and molecular graphs. For example, the toxicity of a compound depends upon the dose and the biological system, and in clinics, it depends upon the clinical information, such as; age, sex, race, and medical history. In other words, the labels are not entirely captured by the structure or any other representation, and the disparity in data labels also exists among the practitioners. The behavior of proteins and compounds can rapidly change in patients, cell lines, and tissues, which may cause a distribution shift. Therefore devising a system, learning the true representation, and labeling the data are major challenges for the success of AI in the drug discovery domain. Many deep learning systems also suffer from repeatability crisis [155] due to stochastic initialization and optimization of parameters, which can be sensitive to the initial settings.

**Figure 9:**
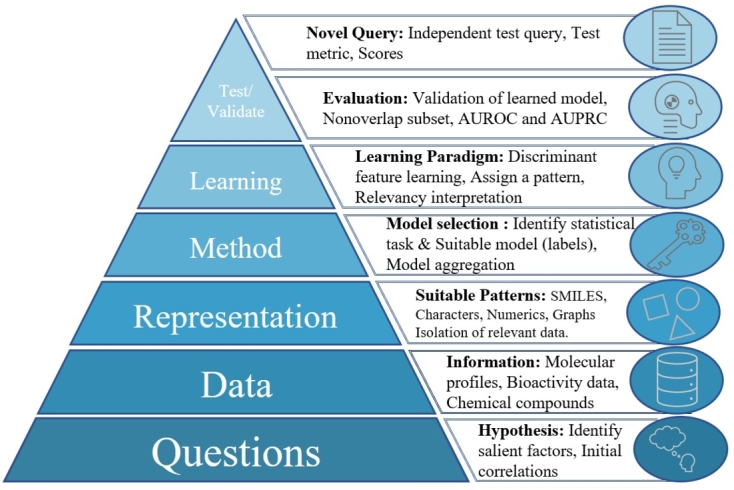
A typical learning pyramid with critical questions that must be kept in mind while developing AI applications for drug discovery.

The type of learning paradigms and evaluation metrics are also important since biological datasets are imbalanced, complex, partially labeled, and not fully understood. Unsupervised or semi-supervised learning can be used to address these challenges and to generate hypotheses for understanding complex diseases and signaling pathways patterns [156]. We also hypothesize that over-fitted machine learning models may generate a novel data-driven hypothesis, which can be validated with experimental Biologists. Reinforcement Learning (RL) can be applied to navigate through the chemical space, which chooses a set of actions to maximize the reward function. RL learning paradigm can be used to generate molecules with desired properties and design optimal treatment strategies. To deal with the imbalanced datasets, we need to obtain data balancing methods, as well as appropriate evaluation metrics. In Fig. 9, we show a pyramid-based learning approach for designing useful AI applications in the drug discovery domain.

AI is very successful in computer vision (CV) and Natural Language Processing (NLP). The problems in CV and NLP are well defined, and the solution is verifiable; such as in face recognition systems, we have the true label to train the system. However, problems related to healthcare are neither well-defined nor verifiable, involving safety and security risks, and leading to privacy concerns. In CV or NLP, a large amount of high-quality labeled datasets are available, and the data can be comprehensively represented in the spatial or temporal domains, relatively easier for a computer program to extract the patterns from the datasets. Whereas, data representation and data labels remain a big challenge in computational chemistry and computational biology. For example; in the chemical domain, there are about 3000 known preset descriptors [157], therefore it is very difficult to say which one captures the most significant data, and how to represent the data for a particular descriptor. Some molecular properties are captured by local features; such as hydrogen bonds or charges, whereas others depend upon external context; ligand binding which is spatially defined.

Another challenge is the black-box nature of deep learning models. We can not fully trust the predictions, without knowing the underlying biological and chemical reasons. We need interpretable and transparent deep learning models and must have a clear grasp of the accuracy of the model, the dynamics of biology, and the precision of our measurements in order to accurately exploit our data [158]. Drug discovery problems have been addressed by black-box optimization methods, such as Bayesian optimization, which find the global optimum (minima or maxima) of a function, to find small molecules that might optimize a specific property [159], or design sequences from initial gene sequences to maximize transcription or translation rates.

### 8.2 Hype

Despite all this progress and investment, only a few AI-based drugs are actually in human clinics [160]. Moreover, the cost of developing a drug is still increasing and there is less adoption of AI tools for clinics at the moment. The pharmaceutical industries are one of the riskiest industry in the world, due to high failure rates and a long timeline. Many traditional drug design scientists still think that all AI-enabled drug development is incremental and hype. The *de novo* design, drug response analysis, molecule optimization, and screening all are stages but most of the drug candidates fail in the clinical trials, making all of the developments incremental. We have a very complex biological space, complex chemical space, and complex clinical space, and optimizing all of them at once is a big challenge.

### 8.3 Hope

MIT Technology Review named the discovery of promising drug-like molecules using AI as one of the top ten technology breakthroughs of 2020 [161]. AI tools have been around for a long time and have shown reasonable success. For example, Wellcome Pharmaceuticals used computational chemistry and modeling to develop the drug Zomig, which is now an approved treatment for migraines [162]. Deep learning was recently used to discover new antibiotics from a pool of 100 million molecules [153]. Insilico Medicine developed Generative Tensorial Reinforcement Learning (GENTRL) AI, a system that can discover and successfully test new compounds in 46 days, making the whole process 15 times faster [163]. The Alliance for AI in Healthcare (AAIH) was founded in September 2018 by several AI companies. In November 2018, AI researchers at Insilico Medicine, led by the AAIH co-founders [164], joined forces to create the ImageNet of generative drug discovery, establishing a set of standards for generative models in healthcare. The success of AlphFold2 is another encouraging example.

In the recent COVID-19 pandemic, AI was used to re-purpose Baricitinib for COVID-19 patients in the United Kingdom (Clinical trial: NCT04421027), which was later validated by the World Health Organization (WHO) [165]. AI also helped in optimizing COVID-19 vaccines [26]. The above precedents of the successful application of AI in the drug discovery process are encouraging and we hope that it will accelerate the role of AI in the drug discovery process.

### 8.4 Reality

AI is most successful in *de novo* molecule design, which is the first stage of the drug discovery pipeline. The next logical step is to check, whether it binds to a target, and check its binding affinity and other properties. Molecular docking, MD simulation, or deep learning can be used for such predictions. These are the chemical stages, where we have a good amount of labeled data for in silico approaches [166]. However, the computer-generated compounds will need to be manually manufactured, evaluated, and optimized at some time.

The more difficult thing is, will the compound produce the same effect *in vivo*? Drugs are chemical compounds, which act on a biological system, which are much more complex and not yet fully understood. Moreover, the clinical patient information further complicates the problem. We have a very large chemical space, an even more, complicated biological space, and the clinical information of patients, which makes it a multi-dimensional difficult learning problem, with fewer data, ground evidence, and unknown labels in many cases. When it comes to AI in drug discovery, what is currently needed is an integrated approach that incorporates both ligand–protein activity and target identification, as well as the compound's properties *in vivo* (pharmacokinetics) with good ground evidence.

The groundbreaking AI system AlphaFold2 predicts the protein structure with high speed and accuracy. However, how to translate this into the *in vivo* situation is still an open question. AlphaFold2 is trained to predict unbound protein structures, whereas most medicinal chemistry applications require protein-small molecule complexes. Secondly, the sub-angstrom resolution is frequently required, which AlphaFold2 cannot provide. Designing protein-based treatments, such as antibodies and peptides, where ultra-high resolution is not required, could be a more successful route for AlphaFold2 [167].

There are also many limitations with AI-based methods in the pharmaceutical industry, such as model interpretation, reproducibility, data access, data labeling, privacy, data quality, and computational infrastructure. Data availability and access are two key components for the success of data science in healthcare. There are also many challenges in statistical learning models. More good-quality datasets with appropriate labels for particular biological questions with suitable representations are needed. Many current data analyses appear to produce very similar results in the end [168], thus, the future of AI in drug discovery is unlikely to lie in the development of the right analysis method, but rather in asking the right question (and thus modeling the right endpoint) in the first place.

It is commonly held in the AI community that we need to collect more and more data, and after that, the data analysis methods can find the activities in the cell or bring new insights, however, this may not be true. Data processing, engineering, and building hypothesis are the key factors in the success of any machine learning algorithm. So, data generated in a hypothesis-free manner will remain difficult to analyze and identify any useful biological or chemical information. Data generated by the push of technology rather than the pull of scientific knowledge need will remain largely useless. Therefore, we need to design algorithms that feed both mind and the machine to adjust their weights and hypothesis. Human physicians will continue making care decisions and treatment strategies, AI can only be used to assist in the decision-making process.

### 8.5 The way forward

**Figure 10:**
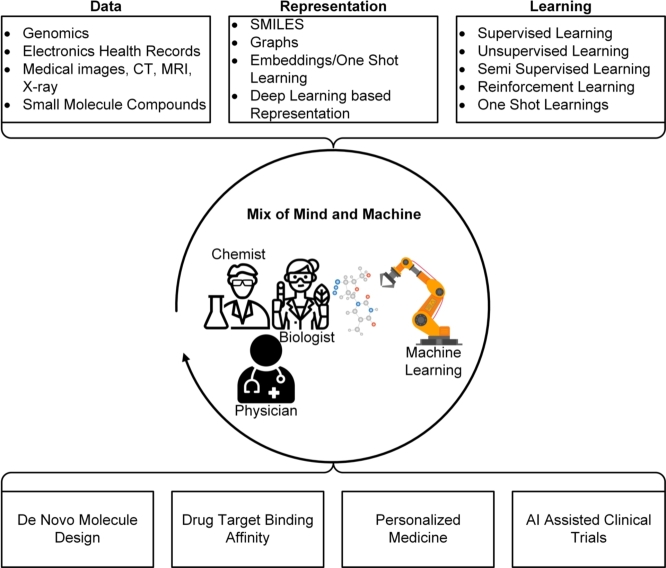
Learning from various data sources can aid drug design, clinical decision support, and public health policy. The collaborative intelligence resulting from the merger of “mind and machine” is expected to improve decision-making in healthcare.

The key to the success of AI in drug discovery is to generate high-quality annotated labeled datasets and learn its representation, which may be possible by collaborative efforts from multiple disciplines. In computer vision, state-of-the-art deep learning models are trained on an ImageNet dataset. We need to develop “ImageNet” for molecules and more benchmarks like MoleculeNet [78]. Robust methods where the human mind can teach the model to optimize so that models generate useful insights that could allow humans to think in new directions are desirable. We need to bring better prospects into clinics, enhance target validation, increase patient recruitment, and improve clinical trial design, as shown in Fig. 10.

Our current AI approach mainly focuses on the manifestations of diseases, rather than the actual causes. Understanding the causal pathway of diseases, through which genetic predisposition may manifest, may enable us to manipulate the disease, as well as reverse the course of the disease. This is a potential venue for causal machine learning. Causal inference [169] can also be used in making treatment decisions and the evolution of patient health.

We also need to cultivate a ‘culture’ among stakeholders, so that they are willing to use computational models and utilize the results. Research and collaboration between industry, academia, and other stakeholders, and the training of professionals to understand both medicine and computer science are needed to fully utilize the potential of data science in the healthcare industry. As an African proverb, “if you want to go fast go alone, and if you want to go far go together”.

More workshops on AI for drug discovery, or computational biology at top AI conferences, like NeuralIPS and ICML should be organized, or perhaps new degree programs for AI in drug discovery are needed in the long-term vision. In 2019, AstraZeneca partnered with Dialog for Reverse Engineering Assessments and Methods (DREAM) to launch a drug-combination challenge on a dataset of 11,576 experiments from 910 combinations across 85 molecularly characterized cancer cell lines. The DREAM Challenges are group competitions that focus on model repeatability and methodological transparency on significant biomedical problems to the scientific community and evaluating participants' forecasts in a statistically rigorous and objective manner. More Competitions like DREAM challenge (http://dreamchallenges.org/) and data analysis competition (CAMDA) [170] and networks (AI3SD) [171], and initiatives like Therapeutics Data Commons [25] are needed to connect experts from different disciplines. More user-friendly machine learning methods, such as AutoML, ClinicalAI and explainable AI (XAI), are needed to enhance the confidence of chemists and doctors for the utilization of machine learning models in daily clinic practice.

## 9 Conclusion

AI-based methods are being adopted in the health care industry where low-cost, intelligent, and flexible methods are affecting areas such as drug design, support for clinical decision making, diagnosis, prevention, and making clinical recommendations [172]. AI applications were previously thought to be inferior to experimental high-throughput screening, combinatorial chemistry, and other technical drivers. It was difficult to create new chemical entities using computer programs, with desired features from the ground up, potentially even better than a human expert [41]. The long and costly process of drug design can be accelerated by employing data science methods for target identification, *De novo* molecular design, drug repurposing, retrosynthesis and prediction of reactivity and bio-activity, FDA approval, and post-market analysis. AI has been implemented by some pharmaceutical organizations, with revenue from AI-based solutions in the pharmaceutical sector estimated to reach US $2.199 billion by 2022 [173].

Deep neural networks (DNNs) can be used to boost prediction power when inferring the properties of small molecules [11], and one-shot learning [174] can be used if a large amount of experimental data is not available. Understanding technical and human errors, labeling constraints, and biological variability associated with the underlying data is crucial to create useful predictive models. It is difficult to represent the experimental data in numerical or computer-assisted form. AI is now being utilized to create representations of trials that allow for data categorization and, ultimately, the development of predictive models [175].

Great things happen in minds and are never done alone, AI is delivering only a platform to execute the plans. We need to develop novel hypotheses for drug discovery by employing the knowledge from different domain experts. After that, we can design a data analysis algorithm, and then we can learn from the data to modulate the hypothesis or modify the algorithms. In short, both mind and machine need to work in synergy. We hope that the use of machine learning, especially deep learning, will increase in the future and help us understand complex biological systems, generate particles with the desired properties, and lead to semi-automated smart healthcare systems. We also expect that AI would be a valuable tool in understanding human biology, a catalyst in combating human diseases and will accelerate drug design. In terms of drug discovery, quality, and safety are more important than speed and cost, devising an AI system that can meet this multi-objective optimization in a multi-dimensional complex space is a huge challenge, which needs collaborative efforts from multiple disciplines in academia and industry.

## Funding statement

The open access publication of this article was funded by the 10.13039/100019779Qatar National Library (QNL), Qatar.

## CRediT authorship contribution statement

Conceived and designed: RQ, TA. Initial draft: RQ, TA. Analyzed and interpreted the data: All authors. Wrote the paper: All authors.

## Declaration of Competing Interest

None declared.
